# The elucidation of plasma lipidome profiles during severe influenza in a mouse model

**DOI:** 10.1038/s41598-023-41055-y

**Published:** 2023-08-30

**Authors:** Marumi Ohno, Siddabasave Gowda B. Gowda, Toshiki Sekiya, Naoki Nomura, Masashi Shingai, Shu-Ping Hui, Hiroshi Kida

**Affiliations:** 1 Division of Biologics Development, International Institute for Zoonosis Control, Hokkaido University, Kita 20 Nishi 10, Kita-ku, Sapporo, 001-0020 Japan; 2 Institute for Vaccine Research and Development (HU-IVReD), Hokkaido University, Sapporo, Japan; 3 Faculty of Health Sciences, Hokkaido University, Kita-12 Nishi-5, Kita-ku, Sapporo, 060-0812 Japan; 4 Graduate School of Global Food Resources, Hokkaido University, Sapporo, Japan; 5 International Collaboration Unit, International Institute for Zoonosis Control, Hokkaido University, Sapporo, Japan; 6 Division of Vaccine Immunology, International Institute for Zoonosis Control, Hokkaido University, Sapporo, Japan

**Keywords:** Infectious diseases, Lipidomics, Inflammation

## Abstract

Although influenza virus infection has been shown to affect lipid metabolism, details remain unknown. Therefore, we elucidated the kinetic lipid profiles of mice infected with different doses of influenza virus A/Puerto Rico/8/34 (H1N1) (PR8) by measuring multiple lipid molecular species using untargeted lipidomic analysis. C57BL/6 male mice were intranasally infected with PR8 virus at 50 or 500 plaque-forming units to cause sublethal or lethal influenza, respectively. Plasma and tissue samples were collected at 1, 3, and 6 days post-infection (dpi), and comprehensive lipidomic analysis was performed using high-performance liquid chromatography–linear trap quadrupole–Orbitrap mass spectrometry, as well as gene expression analyses. The most prominent feature of the lipid profile in lethally infected mice was the elevated plasma concentrations of phosphatidylethanolamines (PEs) containing polyunsaturated fatty acid (PUFA) at 3 dpi. Furthermore, the facilitation of PUFA-containing phospholipid production in the lungs, but not in the liver, was suggested by gene expression and lipidomic analysis of tissue samples. Given the increased plasma or serum levels of PUFA-containing PEs in patients with other viral infections, especially in severe cases, the elevation of these phospholipids in circulation could be a biomarker of infection and the severity of infectious diseases.

## Introduction

Accumulating evidence has revealed the role of lipids in the progression and suppression of diverse diseases. In particular, polyunsaturated fatty acids (PUFAs) have received considerable attention for their pro- and anti-inflammatory actions. Among PUFAs, arachidonic acid is one of the most important lipids because it is a precursor of downstream molecules, such as prostaglandins, leukotrienes, and thromboxanes. These eicosanoids significantly regulate inflammatory responses by causing vasocontraction/vasodilation, leukocyte migration, platelet activation, etc^1^. Arachidonic acid is hydrolyzed from intracellular phospholipids by phospholipases including cytoplasmic phospholipase 2 (cPLA2). Notably, cPLA2 activation by infection with viruses, such as human immunodeficiency virus^2^, human cytomegalovirus^3^, respiratory syncytial virus^4^, and influenza virus^5^, can induce increased production of free arachidonic acid. Since cPLA2 deficiency prevents pneumonia caused by severe acute respiratory syndrome coronavirus (SARS-CoV) infection^6^ and experimental autoimmune encephalomyelitis^7^ in mouse models, the arachidonic acid cascade may be critically involved in the pathogenesis of various inflammatory diseases. Unlike arachidonic acid, docosahexaenoic acid (DHA) and eicosapentaenoic acid (EPA) have been studied for their anti-inflammatory effects; both DHA and EPA decrease the production of inflammatory cytokines and prostaglandins^8^ and inhibit the endotoxin-induced inflammatory cascade^9^.

Because free arachidonic acid is further converted into prostaglandins and other molecules that induce various inflammatory responses, stimulation of arachidonic acid production in infectious diseases has been extensively studied, as described above. In addition, early studies have already demonstrated that viral infections can affect lipid composition, such as the ratios of phospholipids and cholesterol esters to total lipids, in the blood and tissues^10, 11^. Furthermore, our recent study revealed modulation of the signaling pathway of sphingosine-1-phosphate by influenza virus infection^12^. These findings demonstrate that lipid metabolism is one of the target functions significantly affected by viral infections in the host. However, information on the comprehensive and kinetic profiles of lipid molecules during infection is limited. To better understand host responses to viral infections, we applied an untargeted lipid measurement strategy to an established severe influenza mouse model^13^. In the analysis, high-performance liquid chromatography (HPLC)–linear trap quadrupole (LTQ)–Orbitrap mass spectrometry (MS), which enables the simultaneous measurement of multiple lipids from different classes in a small amount of plasma samples^14^, was used. The results demonstrated a unique lipid profile in biological samples from the lethally infected mice.

## Materials and methods

### Chemicals

Liquid chromatography/mass spectrometry (LC–MS) grade methanol, isopropanol, chloroform, and 1 M ammonium acetate solution were obtained from Wako Pure Chemical Industries, Ltd. (Osaka, Japan). The EquiSPLASH Lipidomix quantitative standard (100 µg/mL) and oleic acid-d9 for MS was purchased from Avanti Polar Lipids (Alabaster, AL, USA).

### Virus

The influenza virus A/Puerto Rico/8/34 (H1N1) (PR8) was kindly provided by the National Institute of Infectious Diseases (Tokyo, Japan). The virus was propagated in 10-day-old embryonated chicken eggs at 35 °C for 48 h, and aliquots of the collected allantoic fluids were stored at − 80 °C until further analysis.

### Mice

Male C57BL/6 mice purchased from Hokudo (Sapporo, Japan) were kept in a BSL-2 laboratory at the International Institute for Zoonosis Control, Hokkaido University, under standard laboratory conditions (room temperature, 22° ± 2 °C; relative humidity, 50 ± 10%) and a 12/12 h light/dark cycle. The mice were administered a standard CE-2 chow diet purchased from CLEA Japan (Sapporo, Japan) with water ad libitum. Experiments were performed on 9–12-week-old male mice.

### Virus infection and sample collection

Virus infection and sample collection were performed as previously reported^13, 15^. PR8 virus at 50 or 500 plaque-forming units (PFU) in 50 µL phosphate-buffered saline (PBS) or PBS only (control) were intranasally inoculated into mice under inhalation anesthesia with isoflurane. Body weight was monitored daily. At 1, 3, or 6 days post-infection (dpi), the mice were euthanized by an overdose of isoflurane followed by cervical dislocation, and their blood, lungs, and liver were collected. Blood samples were centrifuged at 2000×*g* for 10 min at room temperature in the presence of heparin sodium (10 U/mL), and supernatants were collected as plasma and stored at − 80 °C until further analysis. Tissues for lipidome analyses were washed in cold PBS to remove excess blood and stored at − 80 °C until sample preparation. Tissue samples for gene expression analyses were homogenized in TRIzol reagent (Thermo Fisher Scientific, Waltham, MA, USA) and stored at − 80 °C until further analyses. Lung samples for virus titer measurement were homogenized in 1 mL RPMI-anti medium [RPMI-1640 (Thermo Fisher Scientific) with 100 U/mL penicillin (Sigma-Aldrich), 100 µg/mL streptomycin (Sigma-Aldrich), and 20 µg/mL gentamicin (Thermo Fisher Scientific)]. After centrifugation at 1750×*g* for 10 min, the supernatants were collected and stored at − 80 °C until further analysis. This study followed the Animal Research: Reporting of In Vivo Experiments guidelines, with the exception of blinding. Because of the requirement to clearly indicate viral infection and treatment with any reagents on the cage cards, the investigators could not be blinded.

### Measurement of virus titers in the lungs

To measure lung virus titers, a plaque assay was performed using lung homogenates, as previously reported^15^.

### Measurement of the levels of cytokines and chemokines in the plasma

The plasma levels of interferon-γ (IFN-γ), interleukin-6 (IL-6), IFN-γ-induced protein-10 (IP-10), monocyte chemoattractant protein-1 (MCP-1), macrophage inflammatory protein-1β (MIP-1β), and tumor necrosis factor-α (TNF-α) were determined using a MAGPIX Milliplex kit (Merck, Darmstadt, Germany) following the manufacturer’s instructions, as reported previously^15^.

### Measurement of selected gene expression

Total RNA was extracted from the tissue samples using TRIzol (Thermo Fisher Scientific, Waltham, MA, USA), and cDNA synthesis was performed using High-Capacity cDNA Reverse Transcription Kits (Thermo Fisher Scientific) following the manufacturer’s instructions. Gene expression of *ethanolamine kinase 1* (*Etnk1*; Mm07299373_m1), (*Ept1*; Mm01210813_m1), *phospholipase A2 group IVA* (*Pla2g4a*; Mm00447040_m1), *acyl-CoA synthetase long-chain family member 4* (*Acsl4*; Mm00490331_m1), and *lysophosphatidylcholine acyltransferase 3* (*Lpcat3*; Mm00520147_m1) was quantified using real-time polymerase chain reaction (PCR) with a StepOne Real-Time PCR System (Applied Biosystems, Foster City, CA, USA) with the indicated TaqMan probes (Applied Biosystems). Gene expression was normalized to 18S (Mm03928990_g1) from the same samples, and relative expression was calculated using the comparative Ct method (ddCt).

### Lipidome analyses

Total lipids were extracted from the liver, lungs, and plasma using Folch’s method with minor modifications^16, 17^. Briefly, weighed liver or lung tissue was transferred into a 1.5 mL Eppendorf tube and homogenized in 10 volumes of methanol and five to six 1.4 mm ceramic beads (Fisherbrand, Pittsburgh, PA, USA) using a Bead Mill 4 (Fisherbrand) homogenizer (30 s, 2 cycles); for plasma samples, 50 µL of plasma was mixed with 100 µL of methanol. Then, 100 µL of the methanolic homogenate containing 10 mg of each tissue or 150 µL of the plasma–methanol mixture was transferred to a new Eppendorf tube, after which 100 µL of a pre-mixed internal standard solution (10 ng of EquiSPLASH + 100 ng of oleic acid-d9) prepared in methanol was added. The mixture was vortexed at 3500 rpm for 30 s. After adding 400 µL of chloroform, the mixture was vortexed at 3500 rpm for 5 min, and 100 µL of Milli-Q water was added with an additional vortex for approximately 30 s. The biphasic extracts were centrifuged at 20,630×*g* for 10 min at 4 °C, and the lower chloroform layer was transferred to a new vial. The lipids contained in the upper aqueous layer were re-extracted with 400 µL of chloroform. The organic extracts were combined, and the organic solvent was evaporated under vacuum. The dried total lipids were re-dissolved in 100 µL of methanol with gentle vortexing and centrifuged at 20,630×*g* for 10 min at 4 °C, after which the supernatant was collected as the LC–MS sample.

The untargeted lipidomic analysis was performed using a prominence UFLC system (Shimadzu Corp., Kyoto, Japan) connected to an LTQ Orbitrap MS (Thermo Fisher Scientific Inc., San Jose, CA) and an Atlantis T3 C18 column (2.1 × 150 mm, 3 µm, Waters, Milford, MA, USA). The flow rate was 200 μL/min with linear flow of the mobile phase, the injection volume via an autosampler was 10 µL, and the column temperature was 40 °C. The mobile phase comprised 10 mM CH_3_COONH_4_ (A), isopropanol (B), and methanol (C) in negative mode with a gradient of 30% B and 35% C (0–1 min); 80% B and 10% C (1–14 min); 85% B and 10% C (14–27 min) or in positive mode with a gradient 6% B and 90% C (0–1 min); 83% B and 15% C (1–10 min); 83% B and 15% C (10–19 min); 6% B and 90% C (19–19.5 min); 6% B and 90% C (19.5–22 min). Mass spectrometric data were acquired in negative mode with electron spray ionization (ESI) under the conditions: capillary temperature (330 °C), sheath gas flow (50 units), and auxiliary gas flow (20 units). The source voltage value was set to 3 kV. The Fourier transform full scan range was set to *m/z* 100–1750 to acquire MS^1^ spectra for high-resolution masses. Tandem MS (MS/MS) was performed at a collision energy of 40 V in the ion-trap mode to obtain low-resolution MS/MS spectra for identifying lipid molecular species. Raw data were processed using MS-DIAL (version 4.2) software (RIKEN, Wako, Japan) for the alignment, identification, and peak processing of lipid species. The semi-quantification of each lipid molecule was performed with the deuterated internal standard of the same lipid sub-classes or representative lipid class category, following the instructions of Lipidomics Standards Initiative (https://lipidomicstandards.org/) level 2 and level 3. The concentration of the lipid molecular species was calculated by taking the peak intensity ratios of each analyte to those of the internal standard and normalized by the weight or volume of the tissue or plasma, respectively. The concentrations of individual lipid molecules in the plasma, liver, and lungs are provided in Supplementary Tables S1, S2, and S3, respectively.

### Plasma lipidome data analysis

The concentrations of 297 molecules were determined in the plasma samples, and the characteristics of lipid profiles in each biological sample were analyzed with MetaboAnalyst 5.0 (http://www.metaboanalyst.ca). Features with more than 25% relative standard deviation were removed (data filtering). The remaining 222 values were then log-transformed and normalized by the autoscaling function. Within them, 169 features were found with false discovery rate (FDR) < 0.01 by one-way analysis of variance (ANOVA). Sparse partial least squares discriminant analysis (sPLS-DA) was performed with 20 variables to calculate variable importance in projection scores for components 1 and 2. Utilizing the top 100 features with lowest FDR values, heatmap analysis with data clustering was performed using Euclidean distance measuring and a Ward clustering algorithm.

### Ethical statement

All mouse experiments were performed with approval (approval# 17-003) from the Animal Care and Use Committee of Hokkaido University following the Fundamental Guidelines for Proper Conduct of Animal Experiment and Related Activities in Academic Research Institutions under the jurisdiction of the Ministry of Education, Culture, Sports, Science, and Technology in Japan. Body weight loss was monitored daily after infection, and mice were humanely euthanized when weight loss reached 25%.

### Statistical analyses

Statistical analyses were performed using Prism 9 software (GraphPad Software, San Diego, CA, USA). Differences were identified using two-way ANOVA or mixed-effects analysis with a correction for multiple comparisons, if necessary, and considered significant when *p* < 0.05. Data are represented as the mean ± SEM.

## Results

### Sublethal and lethal infection with PR8 virus in male C57BL/6 mice

Our previous studies defined severe symptoms of mice infected with the PR8 virus infection at 500 PFU, significant weight loss reaching over 25% at 3–6 dpi, and blood coagulation abnormalities such as increased thrombin production and intravascular blood clotting in the lungs^13, 15^. Therefore, this infection condition was employed to cause lethal disease in mice. In addition, infection at 50 PFU, which does not cause these pathological events, was used as a sublethal disease condition. Based on the observations in the previous studies, the plasma and lungs were collected from mice at 1, 3, and 6 dpi for representing samples at the very early, symptom-onset, and lethal stages during influenza, respectively.

**Figure 1:**
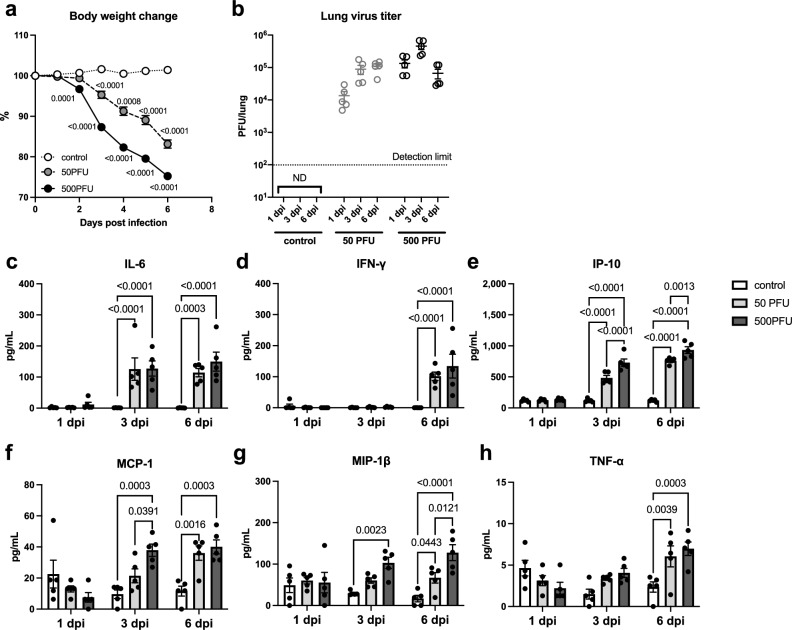
Body weight change, virus titers in the lungs, and plasma concentrations of cytokines/chemokines. Male C57BL/6 mice were intranasally inoculated with PBS control or PBS comprising PR8 virus (50 or 500 PFU), and (**a**) body weight change was monitored. At 1, 3, and 6 dpi, lung and plasma samples were collected to evaluate (**b**) virus titers in the lungs and (**c**–**h**) cytokine/chemokine concentrations in the plasma. (**a**) The body weight change of the mice was calculated as a percentage of the original weight. Symbols represent mean ± SEM (n = 5–15 mice; 5 mice in each group were euthanized at 1, 3, and 6 dpi). White, gray, and black symbols indicate data from PBS control, and 50 or 500 PFU of PR8 virus-infected mice, respectively. Mixed-effect analysis using a multiple comparison correction. (**b**) Virus titers in the lungs were determined by a plaque assay on MDCK cells. Dots represent individual values, and the mean values with SEM are indicated by lines (n = 5). Gray and black symbols indicate data from mice infected with 50 and 500 PFU of the PR8 virus, respectively. (**c–h**) Concentrations of cytokines and chemokines in the plasma, (**c**) IFN-γ, (**d**) IL-6, (**e**) IP-10, (**f**) MCP-1, (**g**) MIP-1β, and (**h**) TNF-α, were determined by a multiplex ELISA kit. In each panel, dots represent individual values, and bars represent the mean ± SEM (n = 5 mice). White, light gray, and dark gray bars indicate data from PBS control, and 50 or 500 PFU of PR8 virus-infected mice, respectively. Two-way ANOVA using a multiple comparison correction. (**a–h**) *PR8* influenza virus A/Puerto Rico/8/34, *PFU* plaque-forming unit, *dpi* day-post-infection, *ND* not detected.

Upon intranasal infection with 50 or 500 PFU of PR8 virus, the mice showed significant body weight loss starting at 3 dpi, whereas the control mice showed no weight loss at any time point (Fig. 1a). Plaque assays were conducted to determine the virus titers in the lungs. In mice infected with 50 PFU, the highest virus titer was observed at 6 dpi, whereas in those infected with 500 PFU, the titer peaked at 3 dpi and decreased at 6 dpi (Fig. 1b). At 3 dpi, the virus titers between the mice in the 500 PFU group was significantly higher than those in the 50 PFU group.

Proinflammatory cytokines and chemokines, IFN-γ (Fig. 1c), IL-6 (Fig. 1d), IP-10 (Fig. 1e), MCP-1 (Fig. 1f), MIP-1β (Fig. 1g), and TNF-α (Fig. 1h), were measured in the plasma samples collected from PR8 virus-infected mice at 1, 3, and 6 dpi. Among them, IFN-γ, IL-6, and TNF-α were similarly increased at 3 and 6 dpi in both infected groups; however, MCP-1, IP-10, and MIP-1β were higher induced in the lethally infected mice than in those infected with a sublethal dose, especially at 3 dpi. These results indicate that viral infection under both conditions caused systemic inflammation, but it was more severe in lethally infected mice than in the sublethal condition. Under these experimental conditions, comprehensive lipidome analyses were further performed to explore lipid profiles associated with influenza virus infection, particularly in the severe case.

### Characterization of the plasma lipid profile during severe influenza

Untargeted lipidome analyses were performed on the plasma samples collected at 1, 3, and 6 dpi by the HPLC–LTQ–Orbitrap MS, and 297 lipid molecular species were identified after confirming their MS/MS spectra. The lipid molecular species from major lipid classes included 148 phospholipids [25 phosphatidylcholines (PC), 50 phosphatidylethanolamines (PE), 2 phosphatidic acids (PA), 6 phosphatidylethanols (PEtOH), 1 phosphatidylmethanol (PmeOH), 17 phosphatidylglycerols (PG), 22 phosphatidylinositols (PI), and 15 phosphatidylserines (PS), 8 cardiolipins (CL) and 2 monolysocardiolipins (MLCL)], 45 lysophospholipids [18 lysophosphatidylcholines (LPC), 13 lysophosphatidylethanolamines (LPE), 1 lysophosphatidic acid (LPA), 2 lysophosphatidylglycerols (LPG), 8 lysophosphatidylinositols (LPI), and 3 lysophosphatidylserines (LPS)], 23 free fatty acids (FAs), 45 sphingolipids [24 ceramides (CER), 7 hexosylceramides (HexCER), and 14 sphingomyelins (SM)], 6 cholesteryl esters (CE), 20 triacylglycerols (TG), and 10 diacylglycerols (DG). Elution profiles and plasma concentrations of the annotated lipids are provided in Supplementary Fig. S1 and Supplementary Table S1. The plasma lipid concentrations of each category are shown in Supplementary Fig. S2. Infected mice demonstrated increased PC, PE, and PG at 3 dpi (Supplementary Fig. S2a), increased lysophospholipids at 3 and 6 dpi (Supplementary Fig. S2b), increased FA at 3 dpi and decreased TG and DG at 3 and 6 dpi (Supplementary Fig. S2c). While increased LPC and LPI, and decreased TG and DG were also observed in sublethally infected mice, the elevation of phospholipids at 3 dpi appeared to be specifically associated with lethal infection.

**Figure 2:**
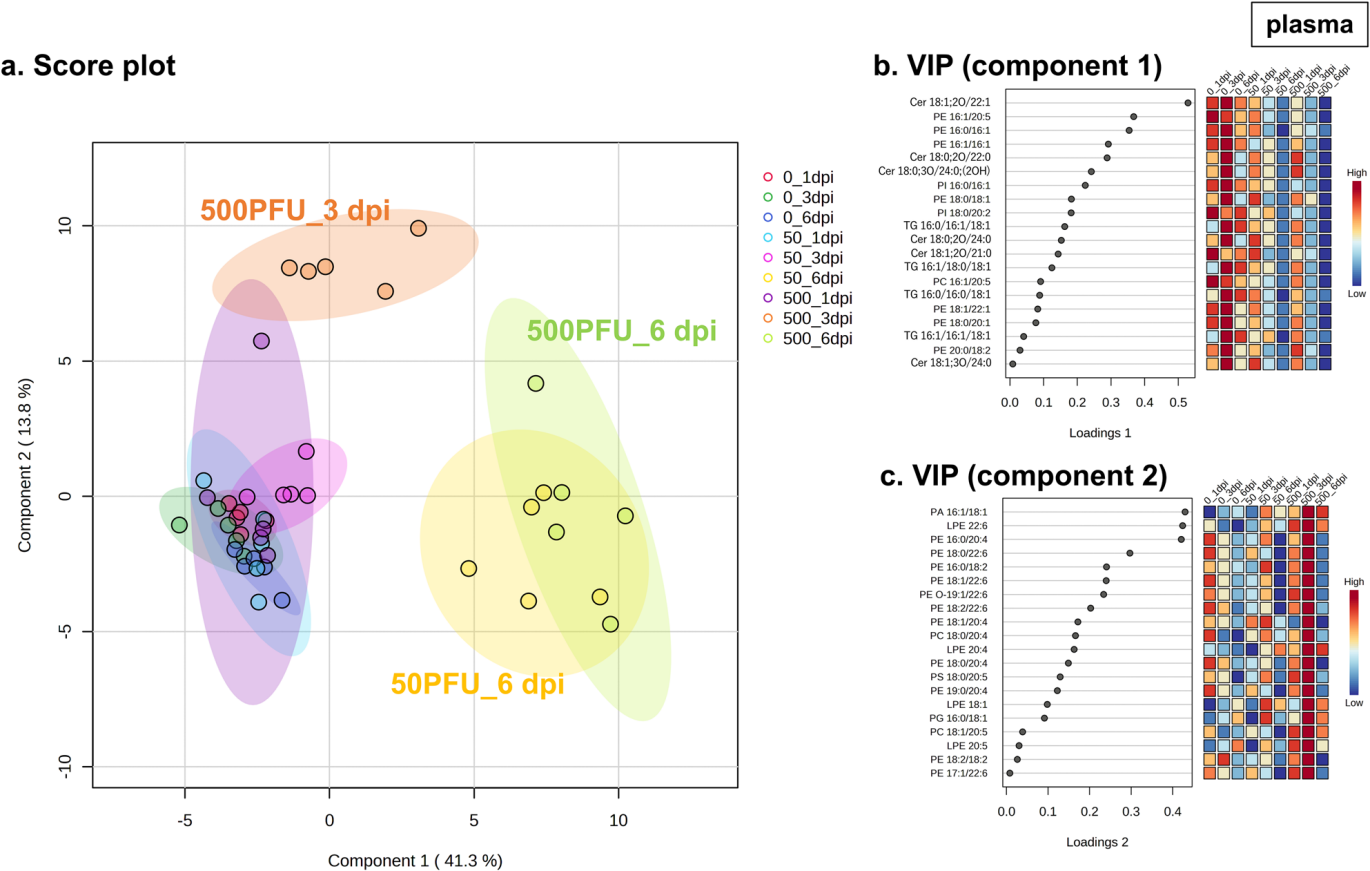
sPLS-DA of plasma lipidome data. Male C57BL/6 mice were intranasally inoculated with PBS control or PBS comprising PR8 virus (50 or 500 PFU), and lipidome analyses determined the concentrations of 297 molecules in the plasma samples collected at 1, 3, and 6 dpi (n = 5 mice). After data filtering on the basis of relative standard deviation, the remaining 222 values were then log-transformed and normalized by the autoscaling function. The sparse partial least squares discriminant analysis (sPLS-DA) was performed with 20 normalized lipid levels in the plasma as variables to calculate variable importance in projection scores for components 1 and 2. (**a**) The score plot between component 1 and component 2. Dots represent samples and circles represent the 95% confidence region for each treatment group. (**b**,**c**) Variable importance for prediction (VIP) scores for (**b**) component 1 and (**c**) component 2. The colored boxes on the right indicate relative values of the features in each group. *PR8* influenza virus A/Puerto Rico/8/34, *PFU* plaque-forming-unit, *dpi* day-post-infection, *PA* phosphatidic acid, *PC* phosphatidylcholine, *PE* phosphatidylethanolamine, *PI* phosphatidylinositol, *PS* phosphatidylserine, *LPE* lysophosphatidylethanolamine, *Cer* ceramide, *TG* triacylglycerol.

Furthermore, sPLS-DA revealed the characteristics of the effect of disease stages and infection conditions, sublethal (50 PFU) and lethal (500 PFU), on the lipid profile in the plasma (Fig. 2a). At 6 dpi, comparable effects of PR8 virus infection under both conditions on plasma lipids were observed, as indicated by similar high values of component 1 scores of the groups in the plots. On the other hand, at 3 dpi, an increase in the component 2 score was observed uniquely in the mice infected under the lethal condition. Top 20 features based on variable importance for prediction (VIP) scores for components 1 and 2 are listed in Fig. 2b and c, respectively, and the individual concentrations of top 5 lipids are shown in Supplementary Fig. S3.

**Figure 3:**
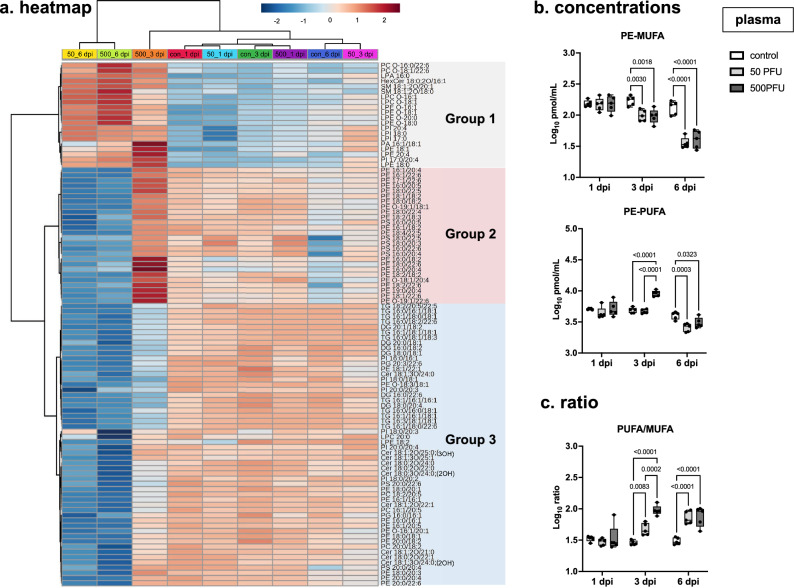
Heatmap analysis of plasma lipidome data. Male C57BL/6 mice were intranasally inoculated with PBS control or PBS comprising PR8 virus (50 or 500 PFU), and lipidome analyses determined the concentrations of 297 molecules in the plasma samples collected at 1, 3, and 6 dpi (n = 5 mice). After data filtering on the basis of relative standard deviation, the remaining 222 values were then log-transformed and normalized by the autoscaling function. Within them, 169 significant features were found; false discovery rate (FDR) < 0.01, one-way analysis of variance (ANOVA). (**a**) Utilizing the top 100 lipids with the lowest FDR values, heatmap analysis with data clustering was performed using Euclidean distance measuring and a Ward clustering algorithm. The colored boxes indicate the relative values of the features in each group. The three major clusters are shown in gray, red, and blue. (**b**) Concentrations of PEs containing monounsaturated fatty acid (MUFA; PE-MUFA) and polyunsaturated fatty acid (PUFA; PE-PUFA) and (**c**) the ratios of PE-PUFA to PE-MUFA are shown separately. (**b**,**c**) In each panel, dots represent individual values, the box shows the interquartile range, the horizontal line within the box shows the median value, and the whiskers/vertical lines show maximum (top) and minimum (bottom) values. White, light gray, and dark gray boxes indicate data from PBS control, and 50 or 500 PFU of PR8 virus-infected mice, respectively. Two-way ANOVA using a multiple comparison correction. (**a**–**c**) *PR8* influenza virus A/Puerto Rico/8/34, *PFU* plaque-forming unit, *dpi* day-post-infection.

The clustering analysis in the heatmap demonstrated that lipid profiles of plasma samples from lethally and sublethally infected mice at 6 dpi were in the same cluster and that lethally infected mice showed a distinct tendency of the profile at 3 dpi (Fig. 3a). In addition to multivariate analysis, univariate analysis on lipidome data also suggested a unique tendency of the lipid profile of lethally infected mice at 3 dpi (Supplementary Fig. S4). Considering the alteration pattern by infection severity and disease stage, lipid molecules were classified to 3 groups; Group 1, increased at 6 dpi under both infection conditions; Group 2, increased uniquely at 3 dpi by lethal infection and decreased at 6 dpi under both conditions; Group 3, decreased under both infection conditions at 6 dpi. While Group 1 mainly consisted of lysophospholipids, most of the lipids in Group 2 were PEs containing polyunsaturated fatty acid (PUFA). Group 3 included TG and DG as well as PE, PC, and PI containing monounsaturated fatty acid (MUFA) containing phospholipids.

### The elevation of PEs containing PUFA in the middle stage of lethal infection

Given the results of sPLS-DA and heatmap analysis, PE molecules were suggested to change significantly by infection depending on infection dose and disease stage. Moreover, the increasing and decreasing tendency of PE appeared to be associated with the degree of unsaturation of the fatty acid contained. The concentrations of MUFA- and PUFA-containing PE (PE-MUFA and PE-PUFA, respectively) are shown in Fig. 3b. The ratio of PE-PUFA to PE-MUFA significantly increased at 3 and 6 dpi, with a higher increase in lethally infected mice compared to those in the sublethal group at 3 dpi (Fig. 3c).

**Figure 4:**
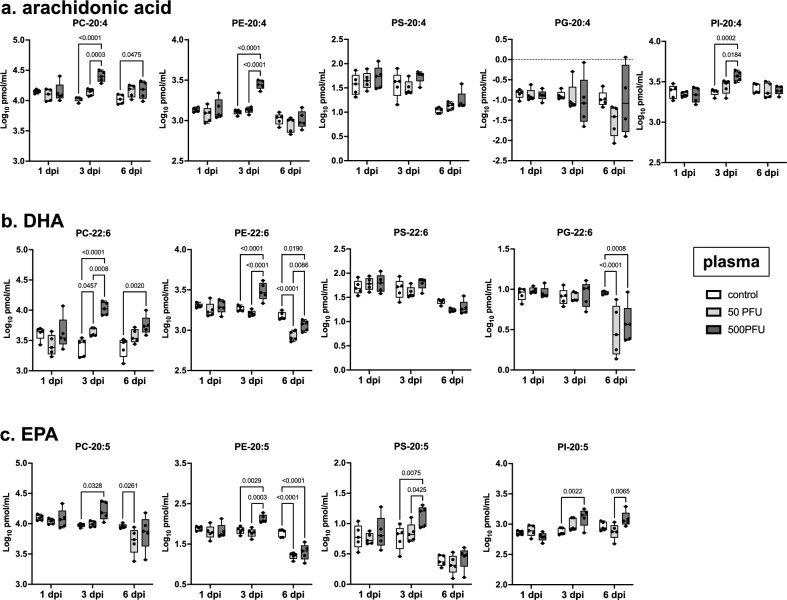
Concentrations of phospholipids containing 20:4, 22:6, or 20:5 in the plasma. Male C57BL/6 mice were intranasally inoculated with PBS control or PBS comprising PR8 virus (50 or 500 PFU), and lipidome analyses were performed with plasma samples collected at 1, 3, and 6 dpi (n = 5 mice). Log-transformed concentrations of phospholipids containing (**a**) 20:4 (arachidonic acid), (**b**) 22:6 (DHA), and (**c**) 20:5 (EPA) in the plasma samples are shown here. In each panel, dots represent individual values, the box shows the interquartile range, the horizontal line within the box shows the median value, and the whiskers/vertical lines show maximum (top) and minimum (bottom) values. White, light gray, and dark gray boxes indicate data from PBS control, and 50 or 500 PFU of PR8 virus-infected mice, respectively. Two-way ANOVA using a multiple comparison correction. *PR8* influenza virus A/Puerto Rico/8/34, *PFU* plaque-forming unit, *dpi* day-post-infection, *DHA* docosahexaenoic acid, *EPA* eicosapentaenoic acid, *PC* phosphatidylcholine, *PE* phosphatidylethanolamine, *PG* phosphatidylglycerol, *PI* phosphatidylinositol, *PS* phosphatidylserine.

Among PE-PUFA, especially arachidonic acid (20:4), DHA (22:6), and EPA (20:5) showed a marked increase in concentrations upon severe infection at 3 dpi, and this trend was also observed in other phospholipids (Fig. 4). The total concentration of PE, PC, and PI containing arachidonic acid in the plasma increased by 2.20-, 2.41-, and 1.59-fold, respectively (Fig. 4a). In addition to arachidonic acid, the concentrations of phospholipids containing DHA were elevated during severe influenza, by 4.23-fold in PC and 1.69-fold in PE (Fig. 4b); PS, PE, PC, and PI containing EPA increased by 2.23-, 1.95-, 1.80-, and 1.77-fold (Fig. 4c). Therefore, an increase in PE and PC containing PUFA, particularly arachidonic acid and DHA, was considered the most prominent feature of the lipid profile during severe influenza.

### Gene expression analyses of enzymes related to PE-PUFA metabolism

**Figure 5:**
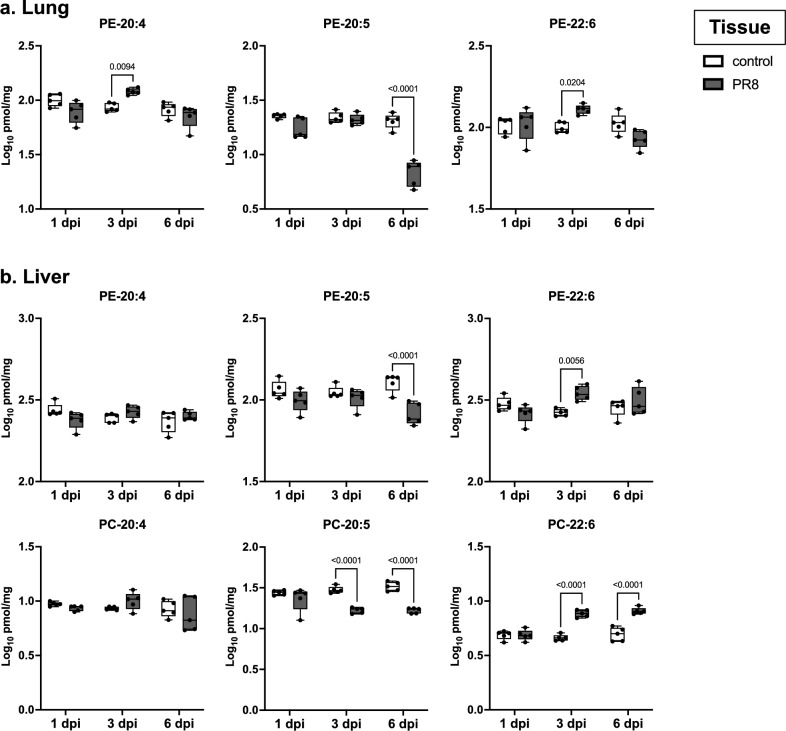
Concentrations of PEs and PCs containing 20:4, 22:6, or 20:5 in the lungs and the liver. Male C57BL/6 mice were intranasally inoculated with PBS control or PBS comprising 500 plaque-forming unit of PR8 virus, and lipidome analyses were performed with tissue samples collected at 1, 3, and 6 dpi (n = 5 mice). Log-transformed concentrations of phosphatidylethanolamines (PEs) and phosphatidylcholines (PCs) containing 20:4 (arachidonic acid), 22:6 (DHA), or 20:5 (EPA) in (**a**) the lungs and (**b**) the liver are shown here. In each panel, dots represent individual values, the box shows the interquartile range, the horizontal line within the box shows the median value, and the whiskers/vertical lines show maximum (top) and minimum (bottom) values. White, light gray, and dark gray boxes indicate data from PBS control, and PR8 virus-infected mice, respectively. Two-way ANOVA using a multiple comparison correction. *PR8* influenza virus A/Puerto Rico/8/34, *dpi* day-post-infection, *DHA* docosahexaenoic acid, *EPA* eicosapentaenoic acid.

To find the source for the PE-PUFA elevated in the plasma, we conducted another infectious experiment with the lethal condition and measured lipid contents in lung and liver samples, which are the infection site and the primary tissue that determines plasma lipid profiles^18^, respectively. Although not all lipid species showed the same trend, in addition to the plasma, the elevation of PE-20:4 and PE-22:6 and that of PE-22:6 and PC-22:6 were confirmed in the lungs and livers, respectively, of severely infected mice at 3 dpi (Fig. 5). Since PC-PUFA was not detected in the lung samples, PE metabolic pathway was further focused on.

**Figure 6:**
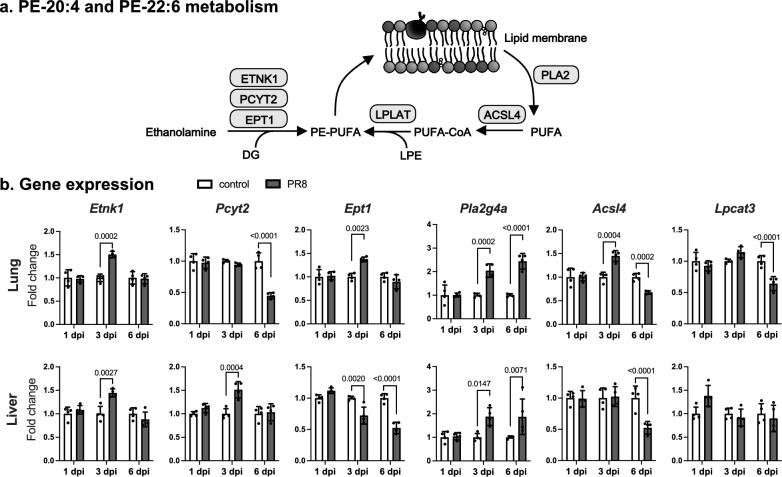
Gene expression of PUFA-containing PE metabolizing enzymes. (**a**) Schematic representation of PE containing PUFA (PE-PUFA) biosynthesis. *PE* phosphatidylethanolamine, *PUFA* polyunsaturated fatty acids, *DG* diacylglycerol, *LPE* lysophosphatidylethanolamine, *ETNK1* ethanolamine kinase 1, *PCYT2* phosphate cytidylyltransferase 2, ethanolamine, *EPT1* ethanolamine phosphotransferase 1, *PLA2* phospholipase A2, *ACSL4* acyl-CoA synthetase long chain family member 4, *LPLAT* lysophospholipid acyltransferase. (**b**) Male C57BL/6 mice were intranasally inoculated with PBS control or PBS comprising 500 plaque-forming unit of PR8 virus, and relative gene expression levels of *Etnk1*, *Pcyt2*, *Ept1*, *phospholipase A2 group IVA* (*Pla2g4a*) encoding cPLA2, *Acsl4*, and *lysophosphatidylcholine acyltransferase 3* (*Lpcat3*) were measured in lung and liver samples collected at 1, 3, and 6 dpi by real-time PCR. Bars represent the mean ± SEM (n = 4 mice). In each panel, dots represent individual values, and white and gray bars indicate data from PBS control and PR8 virus-infected mice, respectively. Two-way ANOVA using a multiple comparison correction on ddCt values. *PR8* influenza virus A/Puerto Rico/8/34, *dpi* day-post-infection.

As shown in Fig. 6a, there are two main pathways for PE-PUFA production; de novo synthesis from ethanolamine and remodeling of PUFA hydrolyzed from lipid membrane. Since viral infection causes the alteration of lipid metabolism^12^, the effect of PR8 virus infection on the gene expression of enzymes related to these pathways was investigated in the lungs and the liver (Fig. 6b). Gene expression levels of *Etnk1* and *Ept1*, rate-limiting enzymes in the reaction of converting ethanolamine to PE, were increased in the lungs at 3 dpi, whereas the liver of infected mice showed the increase and the decrease in those of *Etnk1* and *Ept1*, respectively at 3 dpi. The expression of PC-metabolizing enzymes was also increased at 3 dpi, particularly in the lung (Supplementary Fig. S5).

In the lungs and the liver of mice severely infected with PR8 virus, the gene expression of *Pla2g4a*, which encodes cPLA2, a well-characterized PLA2, that hydrolyses arachidonic acid from the lipid membrane, was significantly increased, suggesting that arachidonic acid hydrolysis from the lipid membrane is facilitated in these tissues upon virus infection. In addition to *Pla2g4a*, *Acsl4* was significantly increased only in the lungs at 3 dpi, whereas no increase in the expression of *Lpcat3*, a well-characterized lysophospholipid acyltransferase (LPLAT) contributing to the production of PE-20:4, PE-22:6, and PC-20:4^19^, was observed. Since ACSL4 critically contributes to the acylation of various PUFA including 20:4, 20:5, and 22:6 in the remodeling pathway of free arachidonic acid^20, 21^, the induction of *Acsl4* observed in the lungs may explain the increase in PE-PUFA at least partially.

## Discussion

In this study, the kinetic lipid profiles during sublethal and lethal influenza in a mouse model were obtained by untargeted lipidome analyses with the HPLC–LTQ–Orbitrap MS, which determined concentrations of 297 lipid molecular species in each plasma sample. Although infected mice showed clear changes in the concentrations of various types of lipids, not all of which may be due to infection. It should be noted that the mice were not starved in this study, so there could be differences in the effects of feeding conditions on lipid profiles between infected and control mice. For example, it is suspected that the decreased TG and DG levels observed in infected mice may have been due to decreased food intake in the animals. This speculation is based on a previous study that most of the lipids including lysophospholipids, phospholipids, CE, and TG were decreased in serum from Sprague–Dawley rats after fasting for 22 h^22^. However, in contrast to simple fasting, influenza virus infection resulted in an increase in lysophospholipids (LPC, LPE, LPG, LPS, and LPI) and phospholipids (PC, PE, PG, and PS) in this study (Supplementary Fig. S2). Particularly, our multivariate and univariate analyses on lipidome data in the plasma demonstrated that the most prominent feature of the lipid profile in the plasma of the lethally infected mice was the elevated concentrations of PC- and PE-PUFA at 3 dpi, when the virus titer in the lungs was the highest. Therefore, the increase in these identified phospholipids is considered to have resulted from lethal infection.

Gene expression analyses demonstrated a significant increase in *Acsl4* as well as PE-biosynthesizing enzymes at 3 dpi in the lungs of infected mice (Fig. 6), which could explain the elevation of PE-20:4 and PE-22:6 in the tissue. A previous study demonstrated higher levels of PE-20:4 and PC-20:4 in the lungs in an influenza ferret model^23^. Although PC-20:4 was reported to be abundant even in the airway cells of uninfected mouse lungs by matrix-assisted laser desorption/ionization (MALDI) imaging MS^24^, it was not detected in our analysis, probably due to differences in lipid detection methods. However, given that ACSL4 is also involved in the PC-20:4 synthesis, and that gene expression of PC-metabolizing enzymes, such as CHK and PCYT1, was significantly increased (Supplementary Fig. S5), PC-20:4 production was thought to be increased in the lungs of infected mice at 3 dpi in the present study. Another possibility is that PC-PUFA abundantly detected in the plasma at 3 dpi was supplied from tissues other than the lung. To address this point, a comprehensive lipidome analysis of plasma and multi-organs from infected mice will be needed. In addition, the critical involvement of ACSL4 in the alteration of phospholipids observed in our study needs to be elucidated in the future, for example by using ACLS4 knockout mice or a specific inhibitor.

At 6 dpi, the expression levels of several enzymes involved in the PE and PC synthesis decreased in the lungs (Fig. 6 and Supplementary Fig. S5), suggesting that phospholipid biosynthesis decreases in the later stage of infection. This notion is supported by the significant decrease in total PEs and PCs in the lungs at 6 dpi (Supplementary Fig. S6), and the same tendency was observed in the plasma (Supplementary Fig. S2). In a previous study, the significant decrease of PC (16:0/16:0), PC (16:0/16:1), PC (16:0/18:2), PE (16:0/18:2), PG (16:0/16:0), and PG (16:0/18:1) was demonstrated in alveolar type II (AT2) cells isolated from influenza virus-infected mice at 6 dpi^25^. All these lipids decreased by 0.47-, 0.39-, 0.69-, 0.76-, 0.33-, and 0.45-fold, respectively, in the lungs of infected mice at 6 dpi also in the present study (Supplementary Table S3). PR8 virus infects and induces cell death in bronchial Clara cells and AT2 cells in mouse lungs^26^. Given that AT2 cells actively produce surfactant lipids^27^ and highly express ACSL4^28^, both infection and infection-induced cell death should have affected lipid metabolism in AT2 cells. Furthermore, since PC (16:0/16:0) and PC (16:0/16:1) are major components of lung surfactant^29^, the significant decrease in the PCs may be associated with the dysregulation of surfactant, which contributes to the host defense against virus infection^30^.

These results suggest that altered gene expression of phospholipid-metabolizing enzymes after infection determined the phospholipid profile in the lungs, which could have affected those in the blood probably through extracellular vesicles secreted from lung cells, such as alveolar epithelial cells, macrophages, and vascular endothelial cells, as reported in lung injury and inflammation^31^. In addition to increased cytokines, the phospholipids increased in the plasma may also transduce inflammatory signals to tissues distant from the site of infection. Interestingly, the liver of infected mice, which is not an infection target of the PR8 virus^32^, also showed an increase of PC- and PE-PUFA, particularly PC-22:6 and PE-22:6, at 3 dpi (Fig. 5). Although the exact mechanisms under which respiratory infection induced these changes need to be elucidated in the future, the altered lipid profiles in extrapulmonary organs may be associated with systemic dysfunction in severe influenza.

The elevation of PC- and PE-20:4 level may be a counteraction against the increase of free arachidonic acid to reduce the production of its metabolites prostaglandins by incorporating the lipid into phospholipids^33^. This is called the arachidonic acid remodeling pathway, where ACSL4 plays a pivotal role. Interestingly, the protein expression of ACSL4 has been reported to be induced by the infection with SARS-CoV-2 and influenza virus^34, 35^, which may explain an increase in PE-20:4 and PE-22:6 reported in the plasma of COVID-19 patients^36^ in addition to that in PE-20:4, PE-22:6, PC-20:4, and PC-22:6 in influenza studies^23^ in animal models including the present study. Clinical studies on patients with Ebola and zika virus infectious diseases also reported elevated PE-PUFA including PE-20:4 and PE-22:6, but not PC-PUFA^37, 38^. This difference may be associated with the tropism of viruses that seems to affect the phospholipid metabolism in each tissue differently. There is a possibility that an increase in PE containing PUFA occurs in various infectious diseases, with induction of ACSL4 as a common mechanism. Although further investigations are needed, PE-PUFA levels in circulation and the ratio of PE-PUFA to PE-MUFA (Fig. 3b) could be a biomarker of diverse infectious diseases, particularly respiratory viral infections.

Furthermore, PE-20:4 and/or its oxidized products may be involved in the pathogenicity of infectious diseases. PC and PE containing PUFA, particularly arachidonic acid and DHA, are highly susceptible to oxidation, causing ferroptosis, a new class of programmed cell death if lipid peroxide reduction is insufficient^20, 39–41^. Given the increase in malondialdehyde or 4-hydroxynonenal, lipid peroxide metabolites, in patients infected with influenza virus^42^, hepatitis C virus^43^, dengue virus^44^, and SARS-CoV-2^45^, the lipid oxidation–reduction balance is thought to incline toward oxidation by viral infections. Notably, direct evidence of phospholipid peroxides-associated cell death has been recently shown in A549 cells infected with influenza virus^46^. Therefore, elucidating the biological significance of altered phospholipid metabolism and oxidized derivatives in the hosts will provide insights into pathogenesis of viral infections.

This study has several limitations. First, it was difficult to compare concentrations between different lipid molecules because relative, but not absolute, quantification was conducted for each lipid. Second, plasma and tissue samples were prepared in different sets of experiments, so that correlation analysis between lipidome data of plasma and tissues could not be performed. In future studies, these points need to be improved.

In summary, we elucidated the plasma lipid profile of mice in sublethal and lethal influenza by untargeted lipidome analyses using HPLC–LTQ–Orbitrap MS. Of note, the increase in plasma concentrations of PE-PUFA was observed only in the middle stage of severe influenza but not in mice with mild disease, when gene expression analyses and lipid profiles in the lungs suggested that the production of PE-PUFA was increased in the lungs. In the future, we will further investigate the biological significance of the alteration in phospholipid metabolism associated with severe influenza in regulating host immune responses, especially immune cell recruitment and infected cell clearance. In addition, the sharp decrease in CE at the early stage of infection in the lungs (Supplementary Fig. S6) is also an attractive research target. These investigations will provide novel insight into the pathogenicity and therapeutic strategy of infectious diseases.

### Supplementary Information

Supplementary Figures.

Supplementary Table S1.

Supplementary Table S2.

Supplementary Table S3.

## Data Availability

The raw data that supports the findings of this study are available on request from the corresponding author.
