# Supplementary material for: The elucidation of plasma lipidome profiles during severe influenza in a mouse model
Source: Sci Rep. 2023 Aug 30;13:14210. doi: 10.1038/s41598-023-41055-y (PMC10469212; doi:10.1038/s41598-023-41055-y)
Supplement: Supplementary file 1 — Supplementary Figures. [file 41598_2023_41055_MOESM1_ESM.pdf]

Title:

The elucidation of plasma lipidome profiles during severe influenza in a mouse model

Authors:

Marumi Ohno[a, b, #], Siddabasave Gowda B. Gowda[c, d, #], Toshiki Sekiya[a, e], Naoki Nomura[a], Masashi Shingai[a, b, e, f], Shu-Ping Hui[b, *], Hiroshi Kida[a, b, *]

Author's affiliation:

[a] Division of Biologics Development, International Institute for Zoonosis Control, Hokkaido University, Sapporo, Japan

[b] Institute for Vaccine Research and Development (HU-IVReD), Hokkaido University, Sapporo, Japan

[c] Faculty of Health Sciences, Hokkaido University, Sapporo, Japan

[d] Graduate School of Global Food Resources, Hokkaido University, Sapporo, Japan

[e] International Collaboration Unit, International Institute for Zoonosis Control, Hokkaido University, Sapporo, Japan

[f] Division of Vaccine Immunology, International Institute for Zoonosis Control, Hokkaido University, Sapporo, Japan

[#] M Ohno and SGB Gowda contributed equally.

*Corresponding author:

Hiroshi Kida

International Institute for Zoonosis Control, Hokkaido University, Kita 20 Nishi 10, Kita-ku, Sapporo 001-0020, Japan

Telephone no.: +81-11-706-9500; Fax no.: +81-11-706-9500.

E-mail address: kida@vetmed.hokudai.ac.jp

*Co-corresponding author:

Shu-Ping Hui

Faculty of Health Sciences, Hokkaido University, Kita-12 Nishi-5, Kita-Ku, Sapporo 060-0812, Japan

Telephone no.: +81-11-706-3693; Fax no.: +81-11-706-3693

E-mail address: keino@hs.hokudai.ac.jp

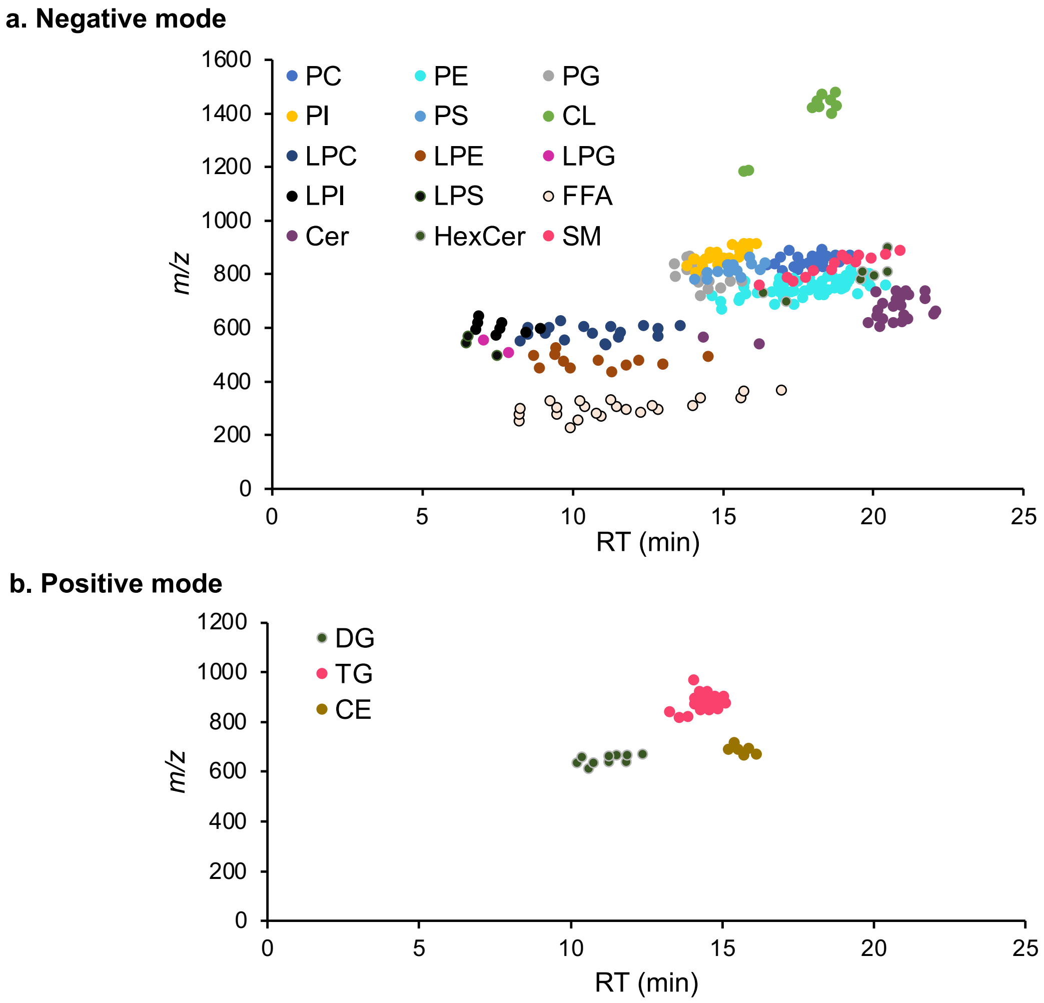

Supplemental Figure S1. Elution profile of the annotated lipids in (**a**) negative and (**b**) positive modes

RT, retention time; PC, phosphatidylcholine; PE, phosphatidylethanolamine; PG, phosphatidylglycerol; PI, phosphatidylinositol; PS, phosphatidylserine; CL, cardiolipin; LPC, lysophosphatidylcholine; LPE, lysophosphatidylethanolamine; LPG, lysophosphatidylglycerol; LPI, lysophosphatidylinositol; LPS, lysophosphatidylserine; FFA, free fatty acid; Cer, ceramide; HexCer, hexosylceramide; SM, sphingomyelin; DG, diacylglycerol; TG, triacylglycerol; CE, cholesteryl ester.

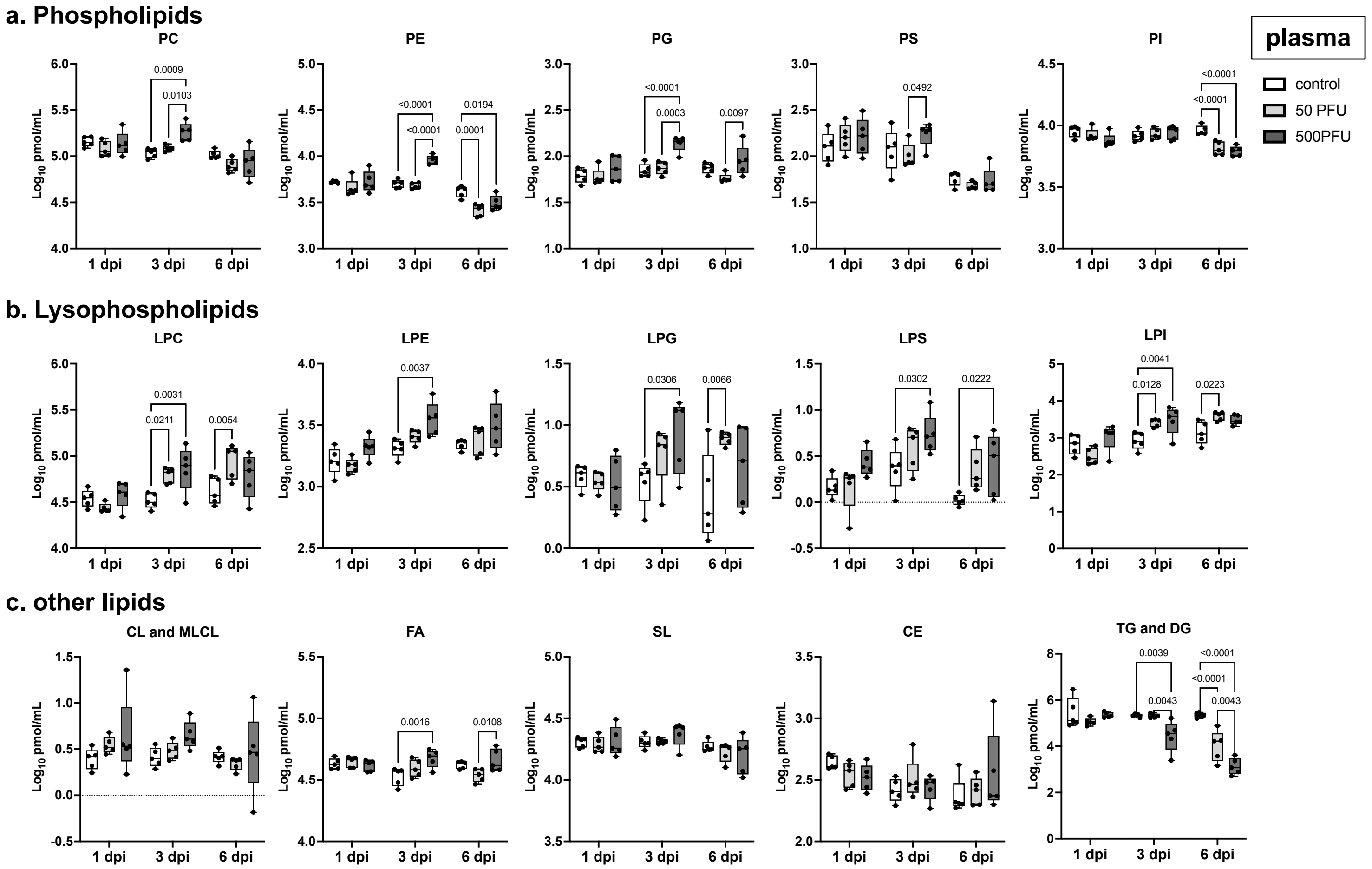

Supplemental Figure S2. Concentrations of lipids in each category in the plasma

Male C57BL/6 mice were intranasally inoculated with PBS control or PBS comprising PR8 virus (50 or 500 PFU), and lipidome analyses were performed with plasma samples collected at 1, 3, and 6 dpi (n = 5 mice). Log-transformed concentrations of (**a**) phospholipids, (**b**) lysophospholipids, and (**c**) other lipids in the plasma samples are shown here. (**a**–**c**) In each panel, dots represent individual values, the box shows the interquartile range, the horizontal line within the box shows the median value, and the whiskers/vertical lines show maximum (top) and minimum (bottom) values. White, light gray, and dark gray boxes indicate data from PBS control, and 50 or 500 PFU of PR8 virus-infected mice, respectively. Two-way ANOVA using a multiple-comparison correction. PR8, influenza virus A/Puerto Rico/8/34; PFU, plaque-forming unit; dpi, day-post-infection; PC, phosphatidylcholine; PE, phosphatidylethanolamine; PG, phosphatidylglycerol; PI, phosphatidylinositol; PS, phosphatidylserine; LPC, lysophosphatidylcholine; LPE, lysophosphatidylethanolamine; LPG, lysophosphatidylglycerol; LPI, lysophosphatidylinositol; LPS, lysophosphatidylserine; CL, cardiolipin; MLCL, monolysocardiolipin; FA, fatty acid; SL, sphingolipid; CE, cholesteryl ester; TG, triacylglycerol; DG, diacylglycerol.

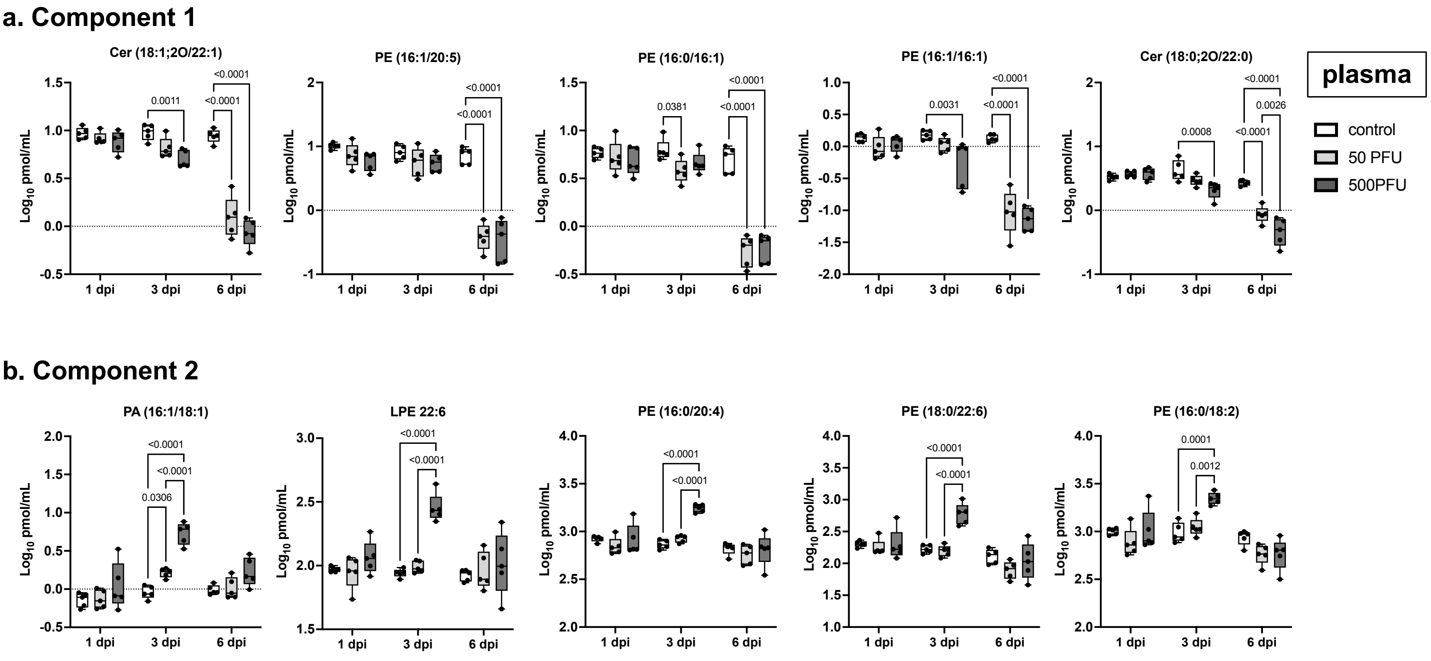

Supplemental Figure S3. Concentrations of top 5 lipids with the highest VIP scores in sPLA-DA

Male C57BL/6 mice were intranasally inoculated with PBS control or PBS comprising PR8 virus (50 or 500 PFU), and lipidome analyses determined the concentrations of 297 molecules in the plasma samples collected at 1, 3, and 6 dpi (n = 5 mice). After data filtering on the basis of relative standard deviation, the remaining 222 values were then log-transformed and normalized by the autoscaling function. The sparse partial least squares discriminant analysis (sPLS-DA) was performed with 20 normalized lipid levels in the plasma as variables to calculate variable importance in projection (VIP) scores for (**a**) component 1 and (**b**) component 2. Log-transformed concentrations of the top 5 lipids in the plasma, with the highest VIP scores. In each panel, dots represent individual values, the box shows the interquartile range, the horizontal line within the box shows the median value, and the whiskers/vertical lines show maximum (top) and minimum (bottom) values. White, light gray, and dark gray boxes indicate data from PBS control, and 50 or 500 PFU of PR8 virus-infected mice, respectively. Two-way ANOVA using a multiple-comparison correction. PR8, influenza virus A/Puerto Rico/8/34; PFU, plaque-forming unit; dpi, day-post-infection; PA, phosphatidic acid; PE, phosphatidylethanolamine; LPE, lysophosphatidylethanolamine; Cer, ceramide.

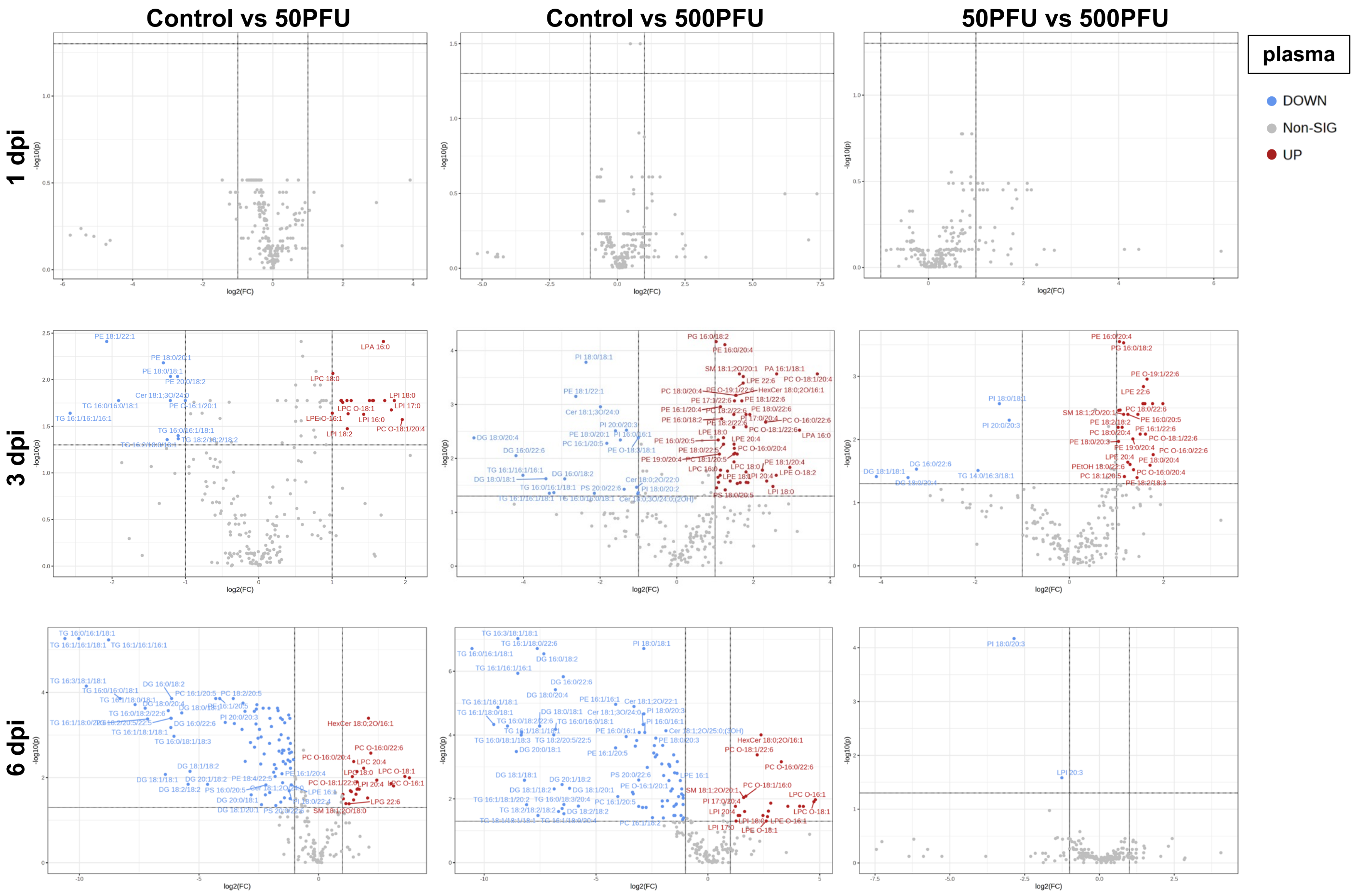

Supplemental Figure S4. Volcano plots

Male C57BL/6 mice were intranasally inoculated with PBS control or PBS comprising 500 plaque-forming units (PFU) of PR8 virus, and lipidome analyses determined the concentrations of 297 molecules in the plasma samples collected at 1, 3, and 6 dpi (n = 5 mice). After data filtering on the basis of relative standard deviation, the remaining 222 values were then log-transformed and normalized by the autoscaling function. Univariate analyses of 3 different infectious condition groups (control, 50 PFU, and 500 PFU) at each time point were performed. Volcano plots indicate lipid molecules that were significantly (FDR adjusted $p < 0.05$) increased [red, $\log_2$(fold-change) > 1] and decreased [blue, $\log_2$(fold-change) < -1]. PR8, influenza virus A/Puerto Rico/8/34; dpi, day-post-infection.

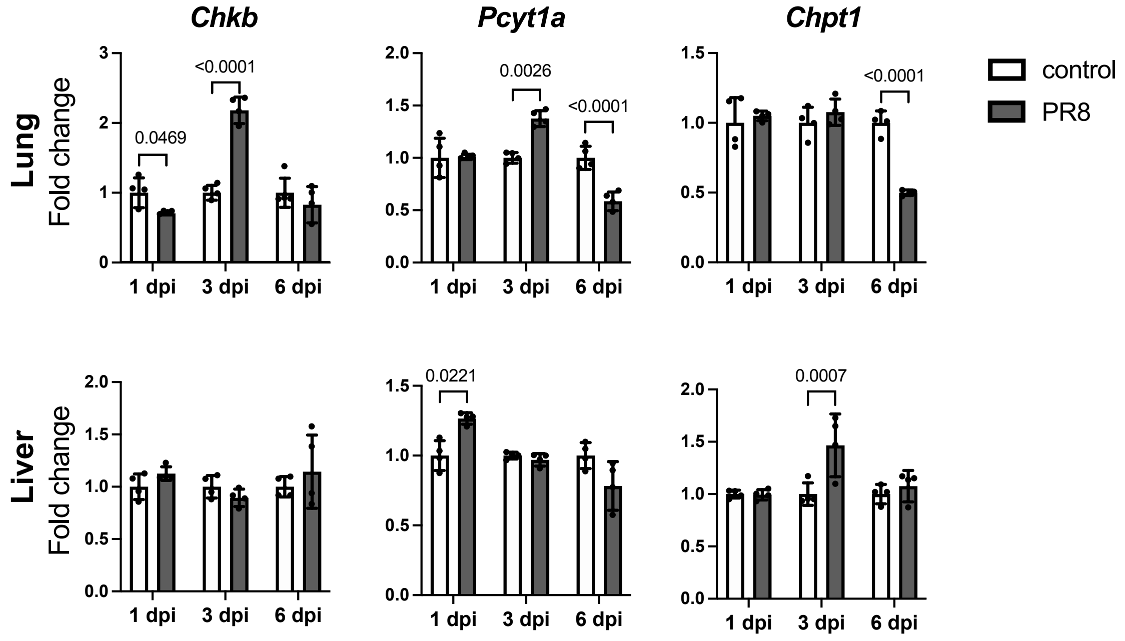

Supplemental Figure S5. Gene expression of PC metabolizing enzymes

Male C57BL/6 mice were intranasally inoculated with PBS control or PBS comprising 500 plaque-forming unit of PR8 virus, and relative gene expression levels of *choline kinase beta* (*Chkb*), *phosphate cytidylyltransferase 1A, choline* (*Pcyt1a*), *cholinephosphotransferase 1* (*Chpt1*) were measured in lung and liver samples collected at 1, 3, and 6 dpi by real-time PCR. Bars represent the mean ± SEM (n = 4 mice). In each panel, dots represent individual values, and white and gray bars indicate data from PBS control and PR8 virus-infected mice, respectively. Two-way ANOVA using a multiple comparison correction on ddCt values. PR8, influenza virus A/Puerto Rico/8/34; dpi, day-post-infection.

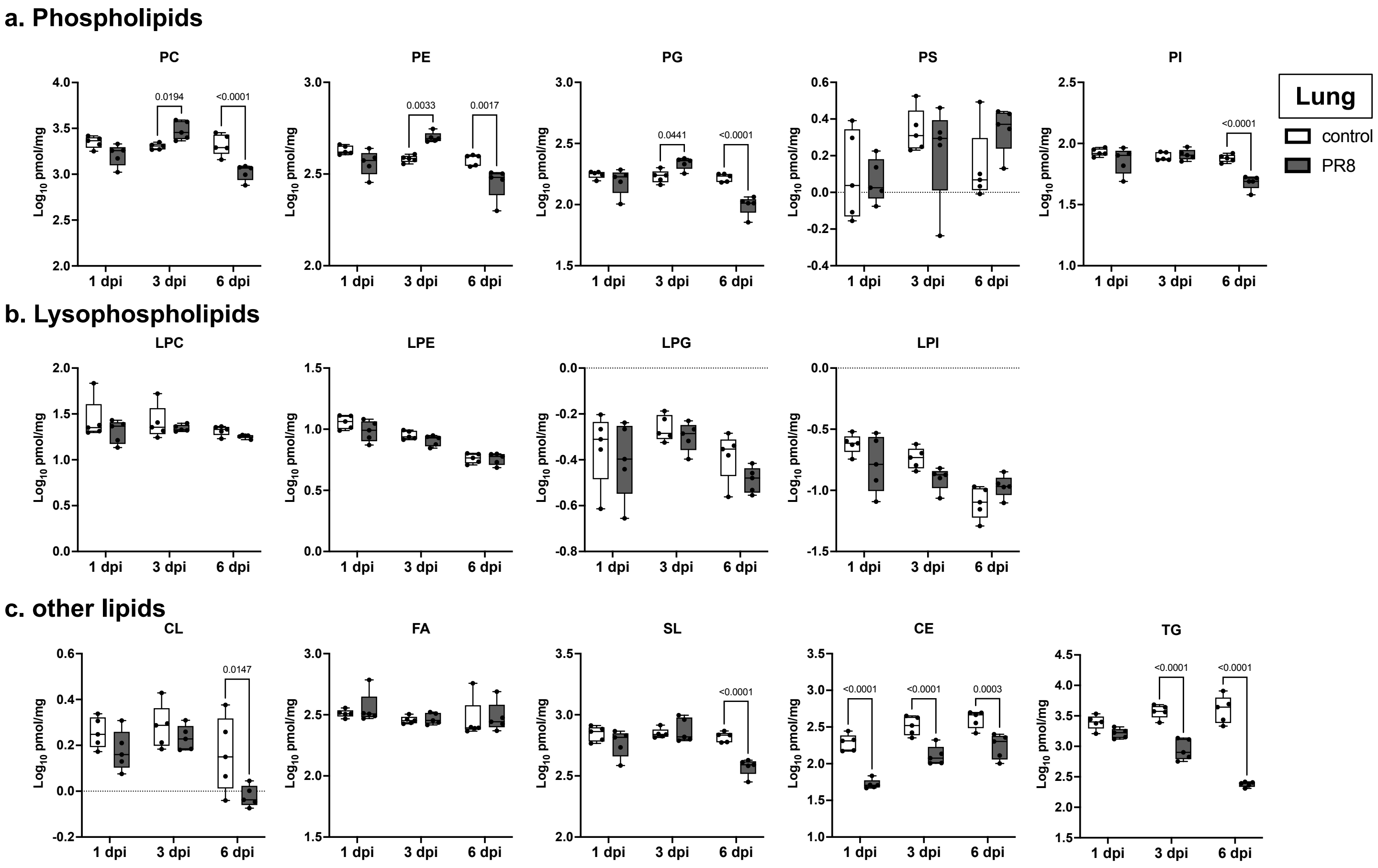

Supplemental Figure S6. Concentrations of lipids in each category in the lung

Male C57BL/6 mice were intranasally inoculated with PBS control or PBS comprising 500 plaque-forming units of PR8 virus, and lipidome analyses were performed with lung samples collected at 1, 3, and 6 dpi (n = 5 mice). Log-transformed concentrations of (**a**) phospholipids, (**b**) lysophospholipids, and (**c**) other lipids in the plasma samples are shown here. (**a**–**c**) In each panel, dots represent individual values, the box shows the interquartile range, the horizontal line within the box shows the median value, and the whiskers/vertical lines show maximum (top) and minimum (bottom) values. White and gray boxes indicate data from PBS control and PR8 virus-infected mice, respectively. Two-way ANOVA using a multiple-comparison correction. PR8, influenza virus A/Puerto Rico/8/34; PFU, plaque-forming unit; dpi, day-post-infection; PC, phosphatidylcholine; PE, phosphatidylethanolamine; PG, phosphatidylglycerol; PI, phosphatidylinositol; PS, phosphatidylserine; LPC, lysophosphatidylcholine; LPE, lysophosphatidylethanolamine; LPG, lysophosphatidylglycerol; LPI, lysophosphatidylinositol; LPS, lysophosphatidylserine; CL, cardiolipin; MLCL, monolysocardiolipin; FA, fatty acid; SL, sphingolipid; CE, cholesteryl ester; TG, triacylglycerol.

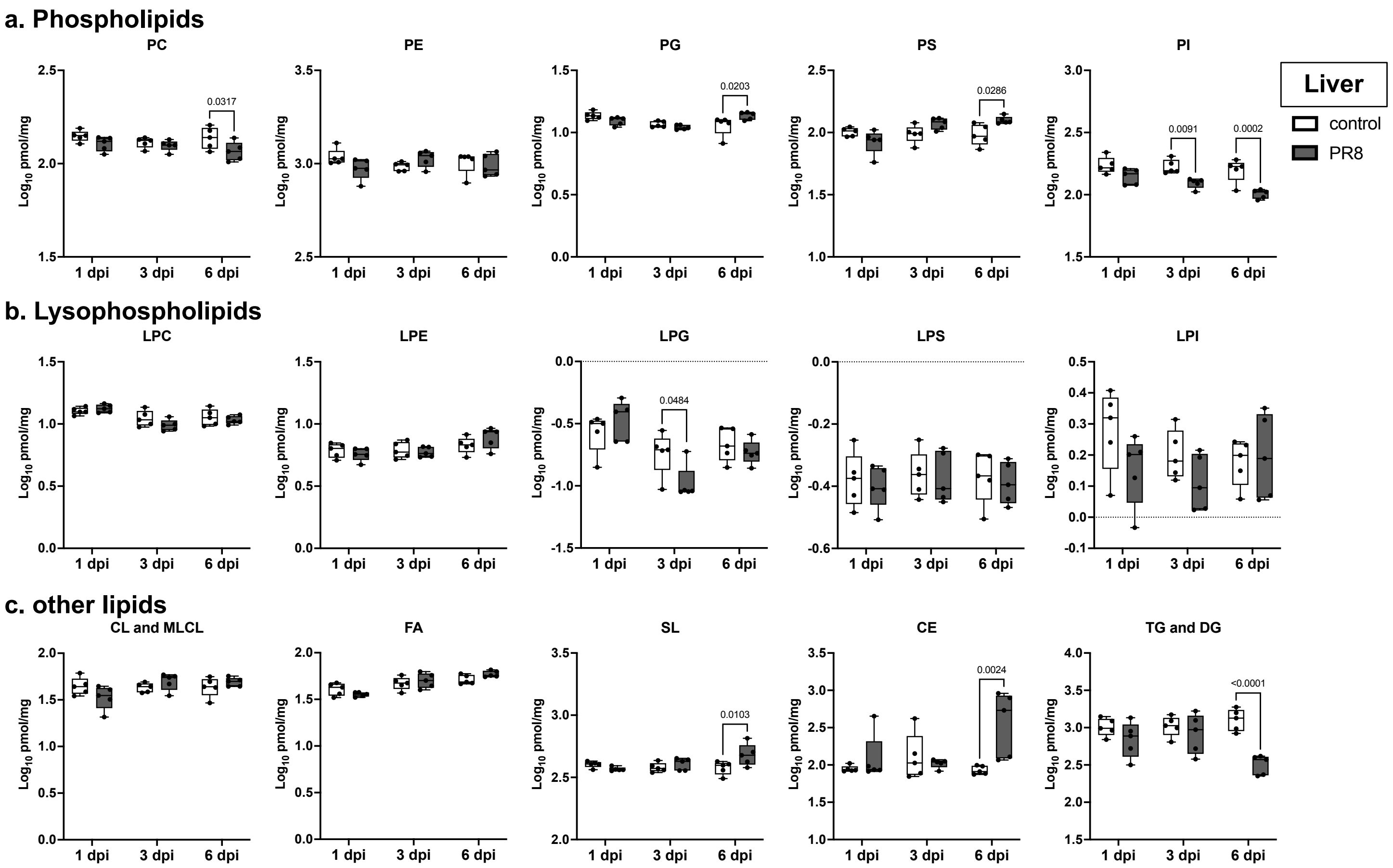

Supplemental Figure S7. Concentrations of lipids in each category in the liver

Male C57BL/6 mice were intranasally inoculated with PBS control or PBS comprising 500 plaque-forming units of PR8 virus, and lipidome analyses were performed with liver samples collected at 1, 3, and 6 dpi (n = 5 mice). Log-transformed concentrations of (**a**) phospholipids, (**b**) lysophospholipids, and (**c**) other lipids in the plasma samples are shown here. (**a**–**c**) In each panel, dots represent individual values, the box shows the interquartile range, the horizontal line within the box shows the median value, and the whiskers/vertical lines show maximum (top) and minimum (bottom) values. White and gray boxes indicate data from PBS control and PR8 virus-infected mice, respectively. Two-way ANOVA using a multiple-comparison correction. PR8, influenza virus A/Puerto Rico/8/34; PFU, plaque-forming unit; dpi, day-post-infection; PC, phosphatidylcholine; PE, phosphatidylethanolamine; PG, phosphatidylglycerol; PI, phosphatidylinositol; PS, phosphatidylserine; LPC, lysophosphatidylcholine; LPE, lysophosphatidylethanolamine; LPG, lysophosphatidylglycerol; LPI, lysophosphatidylinositol; LPS, lysophosphatidylserine; CL, cardiolipin; MLCL, monolysocardiolipin; FA, fatty acid; SL, sphingolipid; CE, cholesteryl ester; TG, triacylglycerol; DG, diacylglycerol.
